# Fabrication of Poly(Ethylene-glycol 1,4-Cyclohexane Dimethylene-Isosorbide-Terephthalate) Electrospun Nanofiber Mats for Potential Infiltration of Fibroblast Cells

**DOI:** 10.3390/polym13081245

**Published:** 2021-04-12

**Authors:** Sofia El-Ghazali, Muzamil Khatri, Mujahid Mehdi, Davood Kharaghani, Yasushi Tamada, Anna Katagiri, Shunichi Kobayashi, Ick Soo Kim

**Affiliations:** 1 Department of Biomedical Engineering, Division of Biomedical Engineering, Faculty of Science and Technology, Shinshu University, Tokida 3-15-1, Ueda, Nagano Prefecture 386-8567, Japan; elghazalisofia@gmail.com; 2 Nano Fusion Technology Research Group, Division of Frontier Fibers, Institute for Fiber Engineering (IFES), Interdisciplinary Cluster for Cutting Edge Research (ICCER), Shinshu University, Tokida 3-15-1, Ueda, Nagano Prefecture 386-8567, Japan; muzamilkhatri@gmail.com; 3 National Engineering Laboratory for Modern Silk, College of Textile and Clothing Engineering, Soochow University, Suzhou 215123, China; mujahid11te83@gmail.com; 4 Department of Calcified Tissue Biology, Graduate School of Biomedical and Health Sciences, Hiroshima University, 1-2-3 Kasumi, Minami-Ku, Hiroshima 734-8553, Japan; Kharaghani66@gmail.com; 5 Faculty of Textile Science and Technology Bioresource and Environmental Science, Shinshu University, Tokida 3-15-1, Ueda, Nagano Prefecture 386-8567, Japan; ytamada@shinshu-u.ac.jp (Y.T.); 19fs703j@shinshu-u.ac.jp (A.K.)

**Keywords:** nanofibers, fibroblast, cell culture, cell adhesion, cell viability, biobased polyester

## Abstract

Recently, bio-based electrospun nanofiber mats (ENMs) have gained substantial attention for preparing polymer-based biomaterials intended for use in cell culture. Herein, we prepared poly(ethylene-glycol 1,4-Cyclohexane dimethylene-isosorbide-terephthalate) (PEICT) ENMs using the electrospinning technique. Cell adhesion and cell viability of PEICT ENMs were checked by fibroblast cell culture. Field emission electron microscope (FE-SEM) image showed a randomly interconnected fiber network, smooth morphology, and cell adhesion on PEICT ENM. Fibroblasts were cultured in an adopted cell culturing environment on the surface of PEICT ENMs to confirm their biocompatibility and cell viability. Additionally, the chemical structure of PEICT ENM was checked under Fourier-transform infrared (FTIR) spectroscopy and the results were supported by -ray photoelectron (XPS) spectroscopy. The water contact angle (WCA) test showed the hydrophobic behavior of PEICT ENMs in parallel to good fibroblast cell adhesion. Hence, the results confirmed that PEICT ENMs can be potentially utilized as a biomaterial.

## 1. Introduction

Recently, bio-based polyesters have been widely studied for biomedical applications such as cell culture, drug delivery, and wound dressing [1,2,3,4,5,6]. Scientists have started exploring the biomedical applications of isosorbide-based polyesters by virtue of their high tensile strength and good thermal stability [4,6,7,8,9]. Electrospun nanofiber mats (ENMs), due to their high surface area, ease of production, fiber network interconnection, biocompatibility, breathability, and resemblance to natural extracellular matrix have been potentially considered for cell culture applications viz cell adhesion, cell migration, and cell viability [10,11,12,13]. In parallel, ENMs have been utilized in several applications including sensors [14], wound dressing [10], drug delivery [5], tissue engineering [15], smart apparels [16,17] high performance filters (water, bacteria, virus, heavy metal, and such impurities) [18,19,20,21]. Depending on the required application, cell adhesion and cell migration behaviors may differ due to the difference in cell size, type, or the culturing surface [4,6]. Scaffolds based on ENMs possess good biodegradability, biocompatibility, non-thrombogenicity, and non-immunogenicity, however, the optimization of such materials is challenging in terms of cell viability, cell infiltration, cell adhesion, and migration properties [4,5,6,10,11,22].

Poly(ethylene-glycol 1,4-Cyclohexane dimethylene-isosorbide-terephthalate) (PEICT) ENMs, in light of their high glass transition temperature [23], good mechanical properties [8], flexibility [6], thermal stability [4], and good crystallinity [23] have potentially been studied for various biomedical applications [4,6].

In our previous report, we investigated pristine poly(1,4-Cyclohexane di-methylene-isosorbide-terephthalate) (PICT), PEICT, and their blended composition. We observed that no sufficient cell infiltration could be achieved due to the unsuitable nanofiber network on the surface, which could not hold breast epithelial cells on the surface of PEICT ENMs [4], therefore, we tried to explore the potential of PEICT ENMs for cell culture applications and to check the effect of surface properties of PEICT ENMs whether they allow fibroblast cell infiltration [4]. Fibroblast cells have been used to check the biocompatibility, cell infiltration, cell adhesion, and cell viability of different polymeric materials [6,13].

To serve the main purpose of this research, isosorbide biobased PEICT ENMs were prepared via electrospinning, and used as a fibroblast cell permeable layer for the first time. There are a few reports that have discussed the chemical structure of PEICT only characterized by FTIR spectroscopy, where in this report, the chemical structure of PEICT ENMs was significantly explored by x-ray photoelectron spectroscopy (XPS) validated by the FTIR results [4,8,23].

The wettability of PEICT ENMs was checked by the WCA test with respect to time as one of the important factors for cell adhesion to optimize the initial resting of fibroblast cells on PEICT ENMs. The nanofiber network and surface topography of PEICT ENMs were studied with respect to fibroblast cell infiltration. The results revealed the potential of PEICT ENM to be used as a biomaterial for fibroblast cell culture.

## 2. Materials and Methods

### 2.1. Materials

The polymer PEICT with an average molecular weight of 46,800 (g/mol) was supplied by SK Chemicals, Gyeonggi-do, Korea [8]. Chloroform (99%), and trifluoroacetic acid (TFA) (98%) were purchased from Wako, Pure Chemical Industries Ltd., Osaka, Japan. Ethanol (99.8%) was obtained by Merck, Japan. Cell migration on PEICT ENMs was performed using NIH 3T3 mouse fibroblast obtained from ATCC (Manassas, VA), USA [24]. The 24-well cell culture flasks and Petri-dishes were obtained from SPL Life Sciences (Pocheon, Korea). Ethylenediaminetetraacetic acid (EDTA) was purchased from Merck (KGaA), Germany. Dulbecco’s modified Eagle’s medium (DMEM) was supplied by Nacalai Tesque, Kyoto, Japan. Fetal bovine serum (FBS) and Dulbecco’s phosphate-buffered saline (DPBS) were provided by HyClone (Logan, UT, USA). The Cell Counting Kit-8 (CCK-8) reagent was obtained from Dojindo Laboratories Japan [25].

### 2.2. Preparation of PEICT ENM

A total of 11% (*w*/*w*) of PEICT solution was stirred at room temperature in chloroform:TFA with a ratio of 3:1 until the formation of a transparent polymer solution. A 10 mL syringe filled with the PEICT polymer solution was installed on an electrospinning power supply (Har-100*12, Matsusada Co., Tokyo, Japan) and supplied with a high voltage of 12.5 kV, keeping a distance of 18 cm from the electrospinning tip to the collector. PEICT ENMs were dried in air at room temperature prior to other necessary characterizations.

### 2.3. Characterizations

The in vitro cytocompatibility of PEICT ENMs was analyzed by NIH 3T3 mouse fibroblast cells, DMEM was used with 10% fetal bovine serum (FBS) incubated at 37 °C with 5% CO_2_. The PEICT ENMs were punched into 3 mm disc-shaped webs and poured into 70% ethanol for sterilization, then washed using PBS after 30 min of dipping. The sterilized PEICT ENMs were placed in a culture flask. Subsequently, each cell culturing well was seeded with NIH 3T3 cells at (1 × 10^3^) density. The cell culture assessment on PEICT nanofibers was carried for one, three, and seven days, where 10 μL WST-1 was added to each flask intermittently after two days. The toxicity, cell proliferation, cell viability, and cell adhesion properties of PEICT ENMs were studied in accordance with previously reported articles [22,24,25].

The morphology of PEICT ENMs was assessed under (field emission electron microscope (FE-SEM; JEOL JSM 6700 F) by JEOL, Japan with the accelerating voltage of (15 kV) and a maximum magnification of 2000×, whereas the morphology of PEICT ENMs before and after cell culture was assessed under SEM (JSM-6010LA) by JEOL, Japan with the accelerating voltage of (10 kV). A fin coater (JFC-1200) by JEOL, Japan was used for coating PEICT ENMs using Pb-Pt for 120 s. The average diameter histogram of PEICT ENMs was calculated using ImageJ software. To investigate the fibroblast cell adhesion and migration on PEICT ENMs, the morphology of fibroblast cells was assessed under scanning electron microscope (SEM) and the images were taken before and after seven days of cell culture. Fibroblast cells, after seven days of culture, were fixed on the PEICT ENMs with 4% of paraformaldehyde for 4 h at 4 °C. PEICT ENMs containing the adhered fibroblasts were dehydrated using ethanol and dried in a vacuum prior to taking images [25].

The chemical composition of PEICT ENMs was assessed under Attenuated Total Reflection Fourier-transform infrared spectroscopy (ATR-FTIR) mode using IR Prestige-21, Shimadzu (Kyoto, Japan). Transmittance spectra were assessed in the wavelength range of 670 to 3000 cm^−1^ at room temperature.

The presence of elements available in the PEICT ENMs was checked under XPS supplied from AXIS Ultra (Schimadzu) with a hemispherical sector analyzer (HSA), dual-anode x-ray source Al/Mg, and the detector. A MgKα x-ray source with 1253.6 eV was used at 1.4 × 10^−9^ torr pressure.

## 3. Results and Discussion

### 3.1. Fibroblast Cell Culture, Cell Adhesion, and Cell Viability on PEICT ENMs

**Figure 1:**
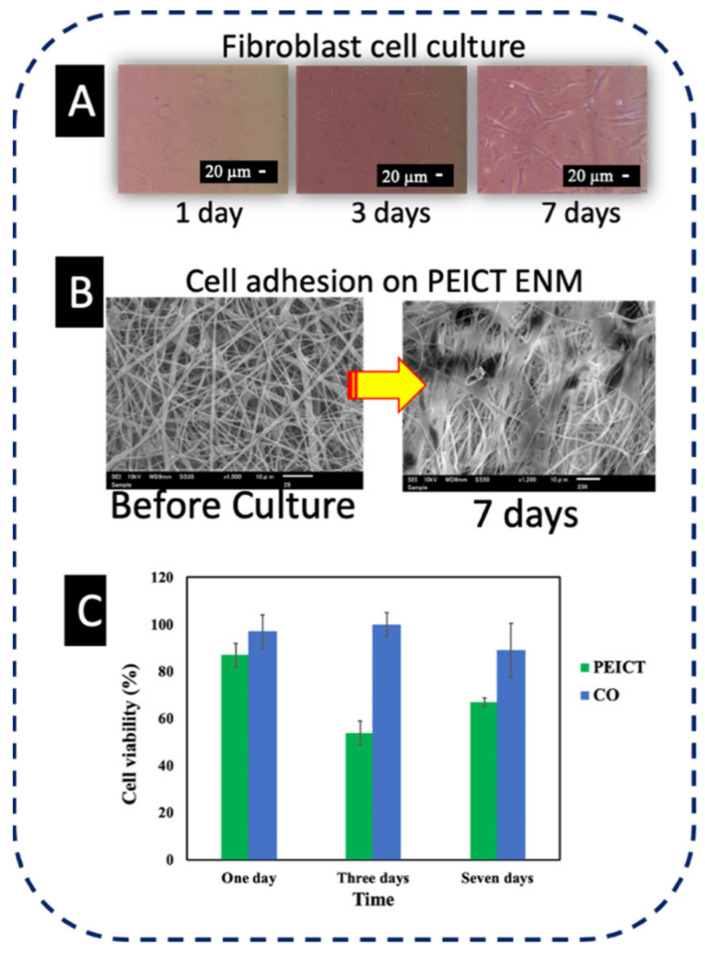
(**A**) Microscopic images of fibroblast cells in the culture flask. (**B**) SEM image of cell adhesion of PEICT ENM. (**C**) Cell viability of PEICT ENMs.

Fibroblast cells were cultured on PEICT ENMs to check the biocompatibility of these nanofibrous mats [22]. Figure 1A shows the microscopic images of the fibroblast cells in the culture flask for one day, three days, and seven days [25]. SEM images were taken after seven days of cell culture on PEICT ENMs, which revealed good cell adhesion as shown in Figure 1B. The reason for this may be the smooth morphology of PEICT ENMs and the optimum cellular infiltration into the porous nanofiber mats. The bar graph shown in Figure 1C reveals cell viability, which demonstrates that the presence of PEICT ENMs in the culturing environment does not have much negative influence on cell growth, where 85–90% of the healthy cells can survive up to one day (until the first day) of culture. After three days of culture, the number of cells was decreased by 35%, but after seven days, the remaining cells showed very good proliferation and the fibroblast cells had multiplied and reached 70% in parallel to very good cell adhesion. These findings suggest that PEICT ENMs can potentially be considered for fibroblast cell culture application.

### 3.2. Morphology of PEICT ENMs

**Figure 2:**
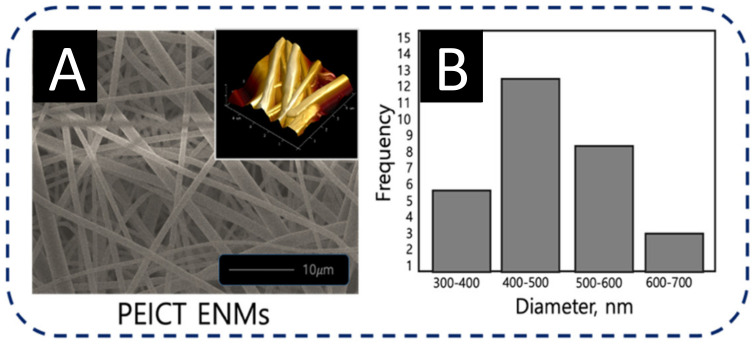
(**A**) FE-SEM image inset with AFM image, and (**B**) average diameter histogram of PEICT ENMs.

To assess the optimized network of nanofibers for the culture and the infiltration of fibroblast cells on the surface of PEICT ENMs, it was necessary to check the morphology and the average fiber diameter of PEICT ENMs. Figure 2A shows the bead-free and the smooth morphology of PEICT nanofibers when electrospun with 11% polymer concentration on a rotating cylinder at a speed of 70 rpm. Figure 2B shows the average diameter distribution graph of PEICT ENMs. The average fiber diameter was found to be 550 ± 60 nm, and the average fiber dimeter distributions of PEICT nanofibers ranging from 300 nm to 700 nm, indicating a non-uniform filtration surface of the PEICT ENM layer, which may be a reason that the infiltration was not 100%.

Compared to the previous reports on PEICT ENMs, this study showed a slightly smaller average fiber diameter size and less diameter distribution as exhibited in the average fiber diameter histogram shown in Figure 2B. In addition, the fiber topography on the surface of PEICT ENM, revealed by the inset atomic force microscopic (AFM) image of Figure 2A, showed a smaller number of gaps between the nanofibers. Good fibroblast cell adhesion on the PEICT ENM surface was perhaps due to the similar frequency of the nanofiber’s average diameter, which helped in providing smoothness in parallel to an interconnected nanofiber network to support fibroblast cell infiltration [4,26,27,28,29].

### 3.3. FTIR Spectroscopy of PEICT ENMs

**Figure 3:**
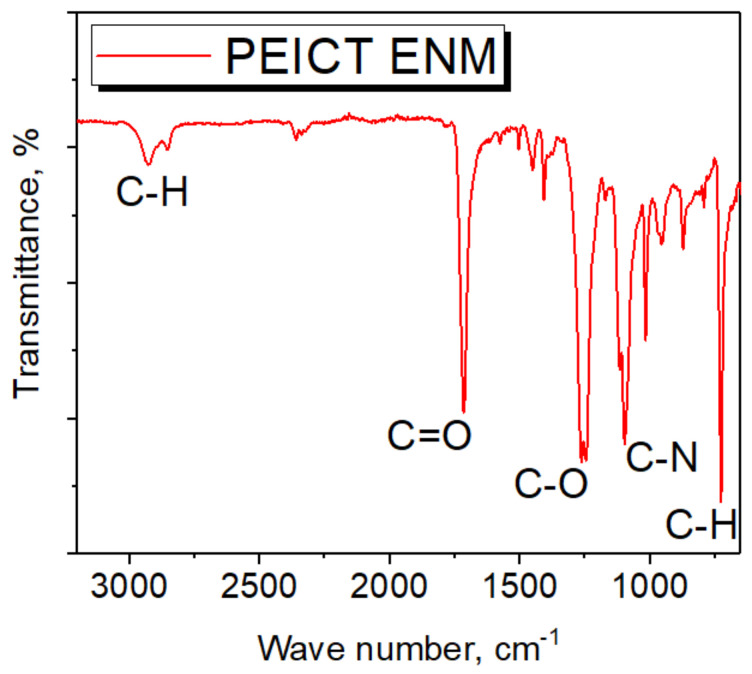
FTIR spectrum of PEICT ENM.

Figure 3 shows the FTIR spectrum of PEICT ENM; the intensive cis peak appearing at 1244 cm^−1^ reveals the ethylene glycol present in its chemical structure, which is in agreement with previous reports [6,8,23]. FTIR spectroscopy further confirms the presence of CH at 2930 cm^−1^ and 726 cm^−1^, C=O at 1715 cm^−1^ and CN at 1094 cm^−1^. The abundance of the –CH group on the surface of PEICT ENM may also be the reason for fibroblast cell growth and cell adhesion [29,30].

### 3.4. XPS Spectroscopy of PEICT ENMs

**Figure 4:**
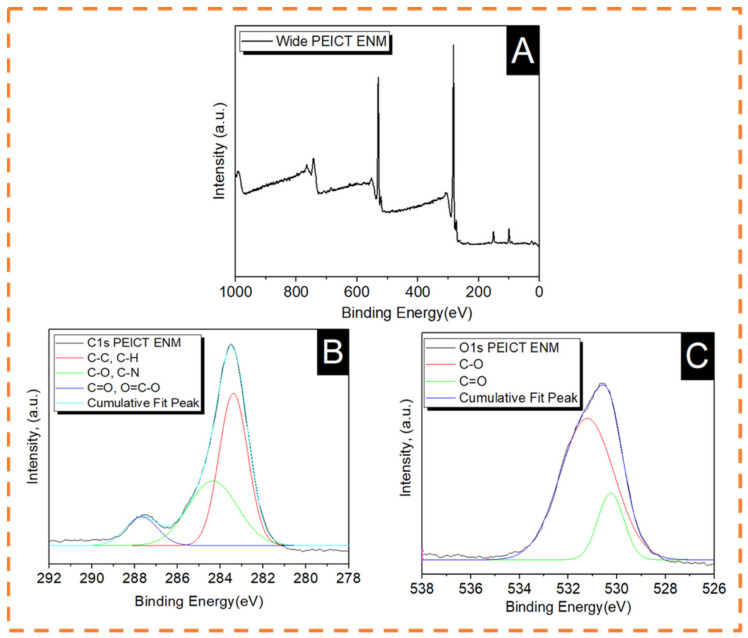
XPS spectra of PEICT ENM. (**A**) Wide spectra. (**B**) Carbon 1s. (**C**) Oxygen 1s.

The main elements present in PEICT ENMs are isosorbide and ethylene glycol, which were previously reported in published articles and revealed by FTIR spectroscopy [4,6,31]. For a significant chemical composition assessment, we chose XPS spectroscopy to deeply understand the chemical composition of PEICT ENMs. Figure 4 shows the XPS spectra of PEICT ENMs. Figure 4A shows the wide XPS spectrum of a PEICT ENM, where two main peaks can be found in the range of Carbon 1s (C1s) and Oxygen 1s (O1s). Therefore, we took XPS spectroscopy of these two intensive regions, C1s ranging from 282 eV to 289.5 eV and O1s ranging from 528 eV and 534 eV, while no intensive peak was observed at N1s due to the small amount of the CN group present in the chemical structure of PEICT ENMs. Figure 4B demonstrates the XPS spectra in C1s, confirming the –CH group abundance on the surface of the PEICT ENM, and also shows the presence of O=C–O, CN, C–C, C=O, and C–O, demonstrating the chemical composition of PEICT ENM more significantly than FTIR and other previous reports [23,31,32,33,34]. The XPS spectra of O1s is given in Figure 4C, indicating C=O and C–O present in the chemical composition of PEICT ENM, which validates the results of C1s and FTIR [34,35].

### 3.5. Wetting Properties of PEICT ENMs

**Figure 5:**
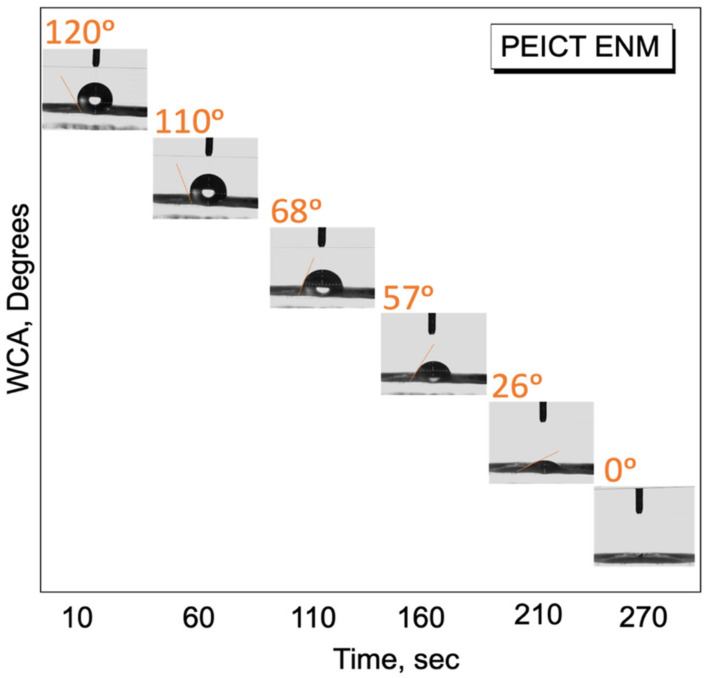
Water contact angle test of PEICT ENMs.

In this report, we cultured fibroblast cells on PEICT nanofibers for the very first time, therefore, it was necessary to check the surface wetting properties as one of the important parameters for cell adhesion [27,30]. PEICT ENMs were assessed by the water contact angle (WCA) test [18,36]. Compared to the previous reports on the WCA of PEICT ENMs (114°) [4], the contact angle observed in this study was slightly higher than 120°, where the obvious reason for this was the similar frequency of the nanofibers’ average diameter, which provided a regular surface with finer fibers that led the water droplet being stable for a certain time prior to starting adsorption on PEICT ENMs. According to Figure 5, the water droplet took about 270 s to reach the angle 0° from 120°, which is in the range of hydrophobic surfaces. Even though the surface of PEICT ENM is hydrophobic [37], the cell infiltration observed was very good because of the interconnected nanofiber network with a smooth morphology, which also played an important role in cell adhesion. Additionally, the WCA test suggested that five minutes were enough as a resting period for fibroblast initial cell adherence on PEICT ENMs.

## 4. Conclusions

PEICT ENMs were successfully electrospun at 11% w/w polymer concentration, the electrospun PEICT nanofibers showed bead-free and smooth morphology with an average diameter of 550 ± 60 nm, having less diameter distributions compared to previous reports on electropun PEICT nanofibers. SEM images showed a good cell viability (70%) and cell adhesion properties on the surface of PEICT ENMs after seven days of cell culture. FTIR and XPS spectroscopies confirmed the chemical structure of PEICT ENMs and the bulk presence of the C–H group on its surface, which was one of the reasons for the good cell adhesion. The results manifested a dependency of the cell infiltration on the fiber network and the average diameter of PEICT ENMs. Ultimately, less gaps on the surface of PEICT ENMs may be a reason for cell infiltration. The WCA test revealed the hydrophobic behavior of the mats with an average WCA of 120 °, which demonstrated the optimum duration of 5 min for the initial resting of cells for their culture prior to starting their culture on PEICT ENMs. Therefore, the results confirmed the potential of PEICT ENMs for fibroblast cell culture application.

## Data Availability

The data presented in this study are available on request from the corresponding author. There is no public repository at our institution.
